# Genomics and proteomics of immune modulatory effects of a butanol fraction of *echinacea purpurea *in human dendritic cells

**DOI:** 10.1186/1471-2164-9-479

**Published:** 2008-10-13

**Authors:** Chien-Yu Wang, Vanisree Staniforth, Ming-Tsang Chiao, Chia-Chung Hou, Han-Ming Wu, Kuo-Chen Yeh, Chun-Houh Chen, Pei-Ing Hwang, Tuan-Nan Wen, Lie-Fen Shyur, Ning-Sun Yang

**Affiliations:** 1 Agricultural Biotechnology Research Center, Academia Sinica, Taipei 115, Taiwan; 2 Institute of Plant and Microbial Biology, Academia Sinica, Taipei 115, Taiwan; 3 Institute of Statistical Science, Academia Sinica, Taipei 115, Taiwan

## Abstract

**Background:**

*Echinacea *spp. extracts and the derived phytocompounds have been shown to induce specific immune cell activities and are popularly used as food supplements or nutraceuticals for immuno-modulatory functions. Dendritic cells (DCs), the most potent antigen presenting cells, play an important role in both innate and adaptive immunities. In this study, we investigated the specific and differential gene expression in human immature DCs (iDCs) in response to treatment with a butanol fraction containing defined bioactive phytocompounds extracted from stems and leaves of *Echinacea purpurea*, that we denoted [BF/S+L/Ep].

**Results:**

Affymetrix DNA microarray results showed significant up regulation of specific genes for cytokines (IL-8, IL-1β, and IL-18) and chemokines (CXCL 2, CCL 5, and CCL 2) within 4 h after [BF/S+L/Ep] treatment of iDCs. Bioinformatics analysis of genes expressed in [BF/S+L/Ep]-treated DCs revealed a key-signaling network involving a number of immune-modulatory molecules leading to the activation of a downstream molecule, adenylate cyclase 8. Proteomic analysis showed increased expression of antioxidant and cytoskeletal proteins after treatment with [BF/S+L/Ep] and cichoric acid.

**Conclusion:**

This study provides information on candidate target molecules and molecular signaling mechanisms for future systematic research into the immune-modulatory activities of an important traditional medicinal herb and its derived phytocompounds.

## Background

*Echinacea *spp., commonly known as purple coneflower, is indigenous to North America. The use of *Echinacea *spp. as an herbal remedy originated in the medicinal culture of North American Indians during the 17^th ^century and was later introduced to Europe. Its use became popular again in the early 1990s and continues today. Currently, *Echinacea *extracts from whole plant or specific tissue (e.g., root or aerial parts) are among the top-selling medicinal or food supplement products in the United States and Europe [1-3]. Recent studies have shown that treatment with specific *Echinacea *extracts activates macrophages, natural killer cells, or other immune cells [4-6]. *Echinacea *extracts have also been reported to stimulate the secretion of cytokines such as tumor necrosis factor-alpha, interferon, interleukin-1 and interleukin-6 [7-10]. *In vivo *studies have shown that treatment with *Echinacea *extracts can increase the number of white blood cells in the circulatory system [11], enhance phagocytosis [12], and trigger the alternate complementary pathway [13]. *Echinacea *extracts have been marketed as possible immune stimulators or enhancers worldwide. These phyto-extracts have been actively evaluated in various clinical studies as candidate therapeutics or preventive remedies for upper respiratory tract infections, common cold, urogenital infection and wound healing [14-19]. However, the results from various studies on the efficacy of *Echinacea *extracts for prevention of experimental colds or common cold have been controversial [16-20]. The most recent study, as a meta-analysis for evaluating the effect of *Echinacea*, addressed again on the potential use and problems of *Echinacea *as remedy for common cold/flu [20,21].

Dendritic cells (DCs) are involved in a spectrum of immune cell functions, including antigen-presentation and phagocytic activity, and play important roles in both innate and adaptive immunities [22]. DCs can capture and transfer molecular or cellular information from the body's outside or interface environment to cells of different immune systems. These cells are not only critical for the induction of primary immune responses but are also involved in the regulation of T cell-mediated immunity [22]. Recently, a series of studies developed DC-based immunotherapy or vaccine protocols designed to elicit specific immunity against certain cancers [12,23,24]. *Echinacea *plant extracts have been shown to have immune-modulatory effects [3,11-13], and we recently reported on the possible use of *Echinacea purpurea *(Ep) phytocompounds as immune-modifiers for human DC activity [25]. *Echinacea *extracts have significant and specific modulatory effects on human DCs, but these effects are plant tissue-specific, the bioactivity varying greatly between root and shoot plus leaf (S+L) tissues. In this follow-up study, we further investigated in detail the effect of a partially purified and chemically defined Ep phytocompound mixture on human DCs.

Recent gene expression profiling in DCs have shown that DCs can actively process environmental signals and activate different transcriptional programs in response to distinct stimuli [26]. In this study, we used functional genomics to analyze changes in gene expression in human immature DCs in response to treatment with the butanol-partitioned fraction (BF) of the S+L tissue extracts of *E. purpurea *[BF/S+L/Ep] and cichoric acid (a major component of this fraction) through Affymetrix gene chip microarray analyses. High-resolution 2-D gel electrophoresis, MALDI-TOF mass spectrometry (MS), tandem MS-MS analysis, and bio-informatics database systems were subsequently employed for proteomics studies in parallel with the genomics studies. Results from these analyses and cell-based bioactivity-guide assays suggest that groups of differentially expressed genes, specific functional genes, and the associated molecular signaling networks can be employed as potential targets for future systematic studies of the response of human DC systems, as a response to traditional herbal medicine formulations or their derived phytocompounds.

## Results

### Effect of [BF/S+L/Ep] extract on expression of cell surface markers in human iDCs

**Figure 1:**
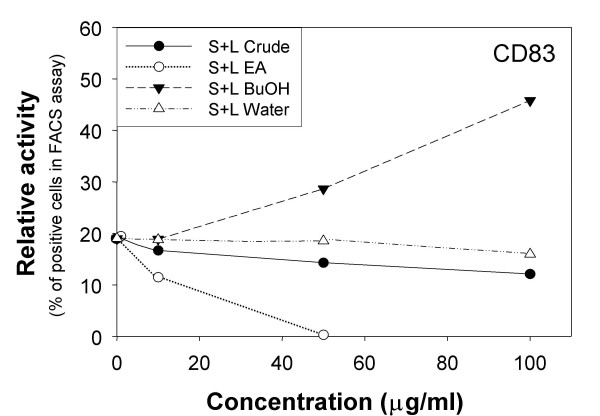
**Specific bioactivities of three subfractions of stem and leaf (S+L) extracts of *E. purpurea *in human immature dendritic cells (iDC).** iDCs were treated for 24 h with the S+L tissue extracts and the derived ethyl acetate (EA), butanol (BuOH), or water fractions. Test cells were subsequently analyzed for cell-surface marker CD83 expression by flow cytometry.

Flow cytometry revealed that [BF/S+L/Ep] enhanced the expression of CD83, a key marker for DC maturation in iDCs, as compared with vehicle (0.1% DMSO) treatment (Figure 1) and the percentage of CD83^+ ^expressing cells increased, from 20% to 45% with 10, 50 and 100 μg/ml [BF/S+L/Ep]. However, treatment with the ethyl acetate (EA) fraction of the S+L extract at 1, 10, or 50 μg/ml significantly decreased the percentage of CD83^+ ^expressing cells from 20% to 0% (Figure 1). The observed effects were not caused by cellular mechanisms related to cytotoxicity, since MTT assay results indicated that treatment with EA (1 to 50 μg/ml) or [BF/S+L/Ep] (1 to 100 μg/ml) showed 98% to 125% cell viability as compared to vehicle treatment (data not shown). Thus, the [BF/S+L/Ep] fraction but not the EA fraction could enhance the maturation of iDCs. To reach this conclusion, it is essential for us to rule out the possibility that lipopolysaccharide (LPS), as a bacterial endotoxin contamination of the [BF/S+L/Ep] extract preparations, might have contributed to the observed results in the present CD83 assay and the subsequent functional genomics and proteomics studies. By using a LAL assay (see Methods), we have obtained a firm negative results (<0.125 EU/ml) on the presence of a significant level of LPS in our [BF/S+L/Ep] extract fraction. EchinaforceTM, a standardized commercial product from Swiss registered *E. purpurea *(L.) Moench fresh plant tincture, was reported to contain an endotoxin level of <0.5 EU/ml, and this tincture was used in a randomized double-blind clinical study [27]. In comparison, our plant extract fraction, [BF/S+L/Ep], was detected to contain a considerably lower level of possible endotoxin contamination (<0.125 EU/ml). We therefore are confident that a LPS effect on various bioactivities can virtually be ruled out from our present studies.

### Compound identification and fingerprint analysis of the bioactive [BF/S+L/Ep] fraction

**Figure 2:**
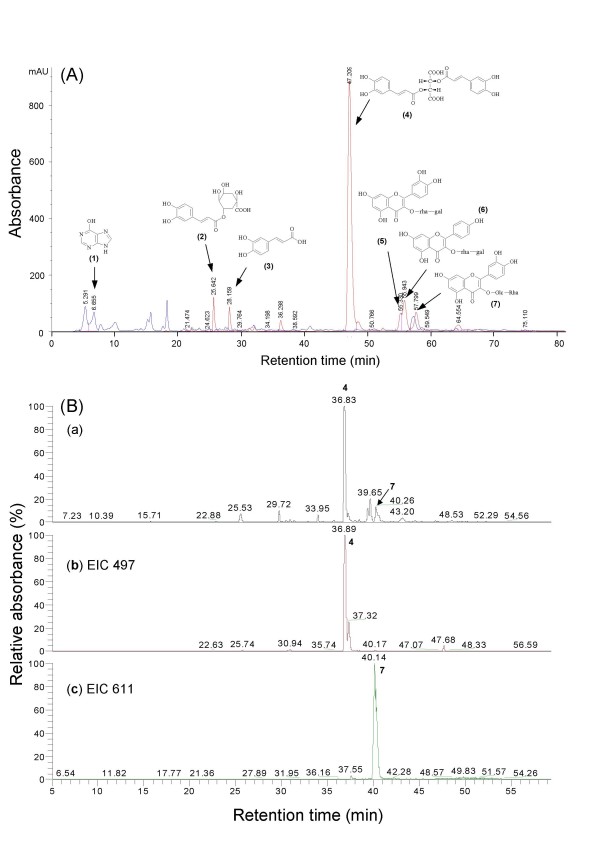
**Chemical fingerprints and candidate index compounds identified in the [BF/S+L/Ep] fraction of *E. purpurea*.** (A) Metabolite profile at 330 and 254 nm and the structures of compounds **1**-**7 **identified from [BF/S+L/Ep]. (B) RP-HPLC chromatogram of [BF/S+L/Ep] at 330 nm (a) analysis on ESI-MS to obtain *m*/*z *ratios of (b) *m*/*z *497, and (c) *m*/*z *611 for compounds **4 **and **7**, respectively.

The overall metabolite profile of [BF/S+L/Ep] of *E. purpurea *was analyzed using RP-HPLC, detected at A_330 _or A_254 _(Figure 2A). Seven phytocompounds (**1**-**7) **namely hypoxanthine (**1**), chlorogenic acid (**2**), caffeic acid (**3**), cichoric acid (**4**), quercetin 3-*O*-rhamnosyl-(1→6)-galactoside (**5**), kaempferol 3-*O*-rhamnosyl-(1→6)-galactoside (**6**), and rutin (**7**) were identified by a combination of chomatography on RP-18 silica gel, Sephadex gel, and C18 RP-HPLC. Structures were elucidated by ^1^H-nuclear magnetic resonance (NMR), ^13^C-NMR and MS, and confirmed with previous data [28-34]. LC/electrospray ionization-MS was further employed to establish the chemical fingerprints with two index compounds of [BF/S+L/Ep]. The RP-HPLC chromatogram detected at 330 nm of [BF/S+L/Ep] was shown in Figure 2B (a) and the mass spectra of the specific compounds were further characterized by using their pseudo molecular weight at *m/z *497 [M + Na]^+^, *T*_*R *_= 36.8 min, compound **4**, Figure 2B (b); and *m/z *611 [M + H]^+^, *T*_*R *_= 40.3 min, compound **7**, Figure 2B (c). These result demonstrate that compounds **4 **and **7 **in [BF/S+L/Ep] can be readily identified by comparing their corresponding peaks (retention time) and MS data.

The levels of cichoric acid (**4**) and rutin (**7**) were quantitatively determined for use as candidate index compounds for the [BF/S+L/Ep] fraction. Standard calibration curves (peak area versus concentrations) prepared for cichoric acid and rutin ranged from 0.125 to 1 mg/ml. Absorbance of the compounds at 330 nm varied linearly within this range. The amount of cichoric acid and rutin were determined as 8.4% (w/w) and 22.3% (w/w), respectively, of the dry weight of the [BF/S+L/Ep] fraction. Together, the results show that this [BF/S+L/Ep] phytocompound mixture was effectively standardized and defined with a specific metabolite profile containing several candidate index compounds (Figure 2). The standardized [BF/S+L/Ep] fraction was then systematically used in the following bioactivity assays to evaluate its immune regulatory effects in human iDCs.

### Affymetrix DNA microarray analysis of differential gene expression patterns in human iDCs in response to treatment with [BF/S+L/Ep] or cichoric acid

**Figure 3:**
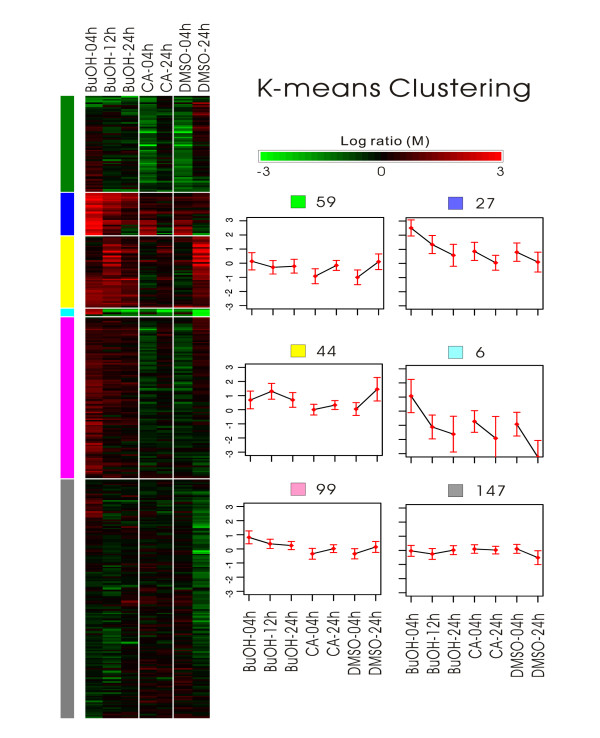
**Comparative analysis of differential gene expression patterns in immature dendritic cells treated with [BF/S+L/Ep] and specific phytocompounds by K-means clustering analyses.** Analysis of test iDC samples (including vehicle control [+DMSO only]) involved comparing the treatment values with untreated values (i.e., zero hour-treated). The left panel shows the heat map of the resulting clustered gene expression. The number of genes involved and the mean profile for each cluster is in the right panel. The mean profile was superimposed with error bars showing ± 1 standard error of the mean.

For DNA microarray analysis, iDCs differentiated from primary monocytes *in vitro *were incubated with [BF/S+L/Ep] or cichoric acid for 4 or 24 h to characterize early- or late-responsive genes. A total of nine oligo-DNA chips were hybridized to determine the transcriptome profiles in human iDCs. The array data was normalized for identifying differentially expressed genes, clustering and annotation. We selected 382 genes for clustering by gap statistic analysis. Figure 3 shows a heat map of differential gene expression patterns and a plot of the resultant 6 possible gene clusters. The K-means clustering revealed that some genes exhibited a consistent response, for example with increased expression at both 4 h and 24 h after treatment with [BF/S+L/Ep]. Alternatively, some genes highly expressed at 4 h but not at 24 h after treatment, or at 24 h but not at 4 h post-treatment, and these may reflect other sets of specific and differential transient gene expression patterns.

Approximately 119 genes were up-regulated by ≥ 2-fold change in expression at 4 h post-treatment with [BF/S+L/Ep], with 20 genes showing 11- to 31-fold change in expression (Table 1A). About 20 genes were down-regulated by > 2-fold change in expression at 4 h post-treatment (Table 1A). Most notably, four up-regulated genes (CCL2 [MCP1], CCL5 [RANTES], NF-κB activator and IL-8), and two down-regulated genes, IFN-α16 and leukocyte immunoglobulin-like receptor [LILRB3], are well known to be involved in various key immune-modulatory activities. Changes in expression noted after 24 h treatment are summarized in Table 1B. Treatment with cichoric acid, a key principle component of the [BF/S+L/Ep] fraction, gave a different expression profile in iDCs. A total of 81 genes and 46 genes were up-regulated and down-regulated, respectively, with 2-fold change in expression at 4 h post-treatment (data not shown). The top 20 up- or down-regulated genes, with 2.6- to 18.0-fold increase or 0.23- to 0.45-fold decrease in expression at 4 h were chosen for further analysis (Table 2A). At 24 h after treatment, 79 genes were up-regulated and 81 down-regulated with ≥ 2-fold change in expression (data not shown). The top 20 up- or down-regulated genes, with 5.36- to 18.9-fold increase or 0.13- to 0.37-fold decrease in expression at 24 h were chosen for further evaluation (Table 2B). As shown in Table 3, among the groups of genes up- or down-regulated by cichoric acid versus [BF/S+L/Ep] treatment for 24 h, an overlapping expression pattern was observed for 31 genes.

**Table 1:** Effect of [BF/S+L/Ep] on gene expression in human iDCs

			**Fold Change**
**Gene Name**	**Common Name**	**Accession No**	**Treatment/Control**
			**(From 31 to 11-fold)**
**(A) Up-regulated at 4 h post-treatment**			
EH-domain containing 1	EHD1	NM_006795	31.81
chemokine (C-C motif) ligand 2	CCL2	NM_002982	22.15
cryptochrome 1 (photolyase-like)	CRY1	NM_004075	21.65
BCL-6 interacting corepressor	BCOR	NM_017745	21.62
tumor necrosis factor receptor superfamily, member 4	TNFRSF4	NM_003327	19.10
Xenopus prevents mitotic catastrophe 2 homolog	XPMC2H	NM_020385	17.54
basic transcription element binding protein 1	BTEB1	NM_001206	17.45
Janus kinase 2 (a protein tyrosine kinase)	JAK2	NM_004972	16.40
wingless-type MMTV integration site family, member 5A	WNT5A	NM_003392	15.42
discs, large (Drosophila) homolog 1	DLG1	NM_004087	15.22
RAB35, member RAS oncogene family	RAB35	NM_006861	14.79
hypothetical protein DKFZp564O0523	DKFZP564O0523	AL136619	14.17
triple functional domain (PTPRF interacting)	TRIO	NM_007118	14.12
zinc finger and BTB domain containing 1	ZBTB1	NM_014950	13.75
chemokine (C-C motif) ligand 5	CCL5	NM_002985	13.62
TRAF family member-associated NF-κB activator	TANK	BC003388	13.12
Interleukin 8	IL8	NM_000584	12.16
HSPCO34 protein	LOC51668	NM_016126	11.37
suppression of tumorigenicity	ST7	NM_013437	11.37
chemokine (C-X-C motif) ligand 2	CXCL2	NM_002089	10.77
**Down-regulated at 4 h post-treatment**			**(From 0.49 to 0.47-fold)**
transducin (beta)-like 2 isoform 1	TBL2	NM_012453	0.50
decorin isoform a preproprotein	DCN	NM_001920	0.50
nucleolar protein family A, member 2	NOLA2	NM_017838	0.50
leukocyte immunoglobulin-like receptor	LILRB3	NM_006864	0.50
calpain 3 isoform a	CAPN3	NM_000070	0.49
interferon, alpha 16	IFNA16	NM_002173	0.49
chromosome 5 open reading frame 4	C5ORF4	NM_016348	0.49
tripartite motif-containing 45	TRIM45	NM_025188	0.49
A kinase (PRKA) anchor protein 7 isoform alpha	AKAP7	NM_004842	0.49
carbonic anhydrase IV precursor	CA4	NM_000717	0.49
uncoupling protein 3 isoform UCP3L	UCP3	NM_003356	0.49
NICE-4 protein	NICE-4	NM_014847	0.48
alpha 2 type V collagen preproprotein	COL5A2	NM_000393	0.48
SWI/SNF-related matrix-associated actin-dependent regulator of chromatin a3	SMARCA3	NM_003071	0.48
CD27-binding (Siva) protein isoform 1	SIVA	NM_006427	0.48
prostate derived STE20-like kinase PSK	PSK	NM_016151	0.48
stanniocalcin 2	STC2	NM_003714	0.48
arginine-glutamic acid dipeptide (RE) repeats	RERE	NM_012102	0.48
stimulated by retinoic acid gene 6	FLJ12541	NM_022369	0.48
zinc finger protein 228	ZNF228	NM_013380	0.48

			**Fold Change**
**Gene Name**	**Common Name**	**Accession No**	**Treatment/Control**
			**(From 28 to 4.6-fold)**

**(B) Up-regulated at 24 h post-treatment**			
protein tyrosine phosphatase, non-receptor type 2	PTPN2	NM_002828	28.77
solute carrier family 26, member 4	SLC26A4	NM_000441	25.07
adaptor-related protein complex 4, sigma 1 subunit	AP4S1	NM_007077	23.91
zinc finger protein RINZF	RINZF	NM_023929	15.88
calbindin 1, 28kDa	CALB1	NM_004929	15.57
chromosome 9 open reading frame 10	C9ORF10	AF214738	12.75
secreted frizzled-related protein 1	SFRP1	NM_003012	10.17
cholinergic receptor, nicotinic, alpha polypeptide 5	CHRNA5	NM_000745	9.95
chorea acanthocytosis	CHAC	AB023203	9.91
WAP four-disulfide core domain 8	WFDC8	AL133571	8.68
tripartite motif-containing 45	TRIM45	NM_025188	8.34
matrix metalloproteinase 2	MMP2	NM_004530	6.73
hook1 protein	HOOK1	NM_015888	6.52
epidermal growth factor receptor pathway substrate 8-related protein 1	EPS8R1	AI343292	6.23
zinc finger protein (ZFD25)	ZFD25	NM_016220	5.27
SUMO-1-specific protease	SUSP1	NM_015571	5.02
nuclear respiratory factor 1	NRF1	NM_005011	4.97
phosphoribosyl pyrophosphate amidotransferase	PPAT	NM_002703	4.86
pleckstrin homology-like domain, family A, member 1	PHLDA1	NM_007350	4.79
vascular Rab-GAP/TBC-containing	VRP	NM_000993	4.61
**Down-regulated at 24 h post-treatment**			**(From 0.03 to 0.24-fold)**
Chromosome 1 open reading fram 29	C1ORF29	NM_006820	0.04
protein (peptidyl-prolyl cis/trans isomerase) NIMA-interacting 1	PIN1	NM_006221	0.04
myxovirus resistance protein 1	MX1	NM_002462	0.09
sialoadhesin precursor	SN	NM_023068	0.10
interferon-induced protein with tetratricopeptide repeats 1	IFIT1	NM_001548	0.12
axonemal dynein heavy chain 7	DNAH7	NM_018897	0.13
ubiquitin specific protease 18	USP18	NM_017414	0.14
regulator of G-protein signaling 12	RGS12	NM_002926	0.14
interferon-induced, hepatitis C-associated microtubular aggregate	IFI44	NM_006417	0.16
secreted protein of unknown function	SPUF	NM_013349	0.17
vacuolar protein sorting 41 (yeast homolog) isoform 1	VPS41	NM_014396	0.18
SP140 nuclear body protein	SP140	NM_007237	0.19
interferon, alpha-inducible protein	G1P2	NM_005101	0.19
interferon-induced protein with tetratricopeptide repeats 4	IFIT4	NM_001549	0.21
small inducible cytokine B11 precursor	CXCL11	NM_005409	0.29
phospholipid scramblase 1	PLSCR1	NM_021105	0.22
viperin	CIG5	NM_080657	0.22
muscle-specific beta 1 integrin binding protein	MIBP	NM_170678	0.23
translation initiation factor IF2	IF2	NM_015904	0.25
serine (or cysteine) proteinase inhibitor, clade A, member 1	SERPINA1	NM_000295	0.25

Top 20 genes up- or down-regulated in human immature DCs in response to treatment with (BF/S+L/Ep) fraction of *Echinacea purpurea *for 4 h (A) or 24 h (B) by fold change in expression.

**Table 2:** Effect of cichoric acid on gene expression in human iDCs

			**Fold Change**
**Gene Name**	**Common Name**	**Accession No**	**Treatment/Control**
			**(From 18 to 2.6-fold)**
**(A) Up-regulated at 4 h post-treatment**			
discs, large (Drosophila) homolog 1	DLG1	NM_004087	18.24
pre-B-cell leukemia transcription factor 2	PBX2	NM_002586	6.45
inhibitor of DNA binding 4, dominant negative helix-loop-helix protein	ID4	NM_001546	4.22
contactin associated protein-like 2	CNTNAP2	NM_014141	4.00
DEAD/H (Asp-Glu-Ala-Asp/His) box polypeptide 6 (RNA helicase, 54 kDa)	DDX6	NM_004397	3.74
guanine nucleotide binding protein (G protein), alpha inhibiting activity polypeptide 1	GNAI1	NM_002069	3.66
tropomodulin 3 (ubiquitous)	TMOD3	NM_014547	3.60
tissue factor pathway inhibitor (lipoprotein-associated coagulation inhibitor)	TFPI	NM_006287	3.13
tolloid-like 1	TLL1	NM_012263	3.09
RAB3 GTPase-activating protein	RAB3GAP	XM_040048	3.08
histone 1, H2al	HIST1H2AL	NM_003511	3.07
solute carrier family 4, sodium bicarbonate cotransporter, member 7	SLC4A7	NM_003615	3.00
fibulin 1	FBLN1	NM_006486	2.97
cytochrome P450, family 2, subfamily C, polypeptide 8	CYP2C8	NM_030878	2.86
peptidyl-prolyl isomerase G (cyclophilin G)	PPIG	NM_004792	2.80
Nijmegen breakage syndrome 1 (nibrin)	NBS1	AK001017	2.76
NADH dehydrogenase (ubiquinone) 1 beta subcomplex, 2, 8 kDa	NDUFB2	NM_004546	2.71
cytosolic ovarian carcinoma antigen 1	COVA1	NM_006375	2.69
zinc finger protein 305	ZNF305	NM_014724	2.67
platelet-activating factor acetylhydrolase, isoform Ib, beta subunit 30 kDa	PAFAH1B2	NM_002572	2.60
**Down-regulated at 4 h post-treatment**			**(From 0.23 to 0.45-fold)**
glycerol kinase	GK	NM_000167	0.23
SMC4 structural maintenance of chromosomes 4-like 1 (yeast)	SMC4L1	AL136877	0.31
ATPase, Ca^+2 ^transporting, type 2C, member 1	ATP2C1	AB037768	0.36
megakaryocyte-associated tyrosine kinase	MATK	NM_002378	0.37
ubiquitin specific protease 24	USP24	XM_165973	0.37
SEC24 related gene family, member D (*S. cerevisiae*)	SEC24D	NM_014822	0.38
talin 1	TLN1	NM_006289	0.39
methionyl aminopeptidase 2	METAP2	NM_006838	0.39
protein kinase, cAMP-dependent, regulatory, type II, beta	PRKAR2B	NM_002736	0.42
endothelial differentiation, G-protein-coupled receptor, 2	EDG2	NM_001401	0.42
integrin, beta 8	ITGB8	NM_002214	0.42
uridine monophosphate synthetase	UMPS	NM_000373	0.42
ribosomal protein L13	RPL13	AA789278	0.43
DNA cross-link repair 1B	DCLRE1B	NM_022836	0.43
chloride channel, calcium activated, family member 2	CLCA2	NM_006536	0.43
caspase 2, apoptosis-related cysteine protease	CASP2	NM_032982	0.44
apoptotic chromatin condensation inducer in the nucleus	ACINUS	NM_014977	0.45
peptidoglycan recognition protein-I-beta precursor	PGLYRPIBETA	NM_020393	0.45

			**Fold Change**
**Gene Name**	**Common Name**	**Accession No**	**Treatment/Control**
			**(From 18.9 to 5.4-fold)**

**(B) Up-regulated at 24 h post-treatment**			
Zinc finger protein 229	ZNF228	AC084239	18.93
adaptor-related protein complex 4, sigma 1 subunit	AP4S1	NM_007077	14.10
chromosome 8 open reading frame 17	C8ORF17	NM_020237	12.13
DKFZP566K023 protein	DKFZP566K023	NM_015485	11.07
glycine receptor, alpha 1	GLRA1	NM_000171	10.81
frizzled homolog 3 (Drosophila)	FZD3	NM_017412	10.31
secreted frizzled-related protein 1	SFRP1	NM_003012	10.11
chorea acanthocytosis	CHAC	AB023203	9.56
actin, alpha, cardiac muscle	ACTC	NM_005159	9.51
tripartite motif-containing 45	TRIM45	NM_025188	8.26
phosphodiesterase 4D, cAMP-specific	PDE4D	AF012073	8.23
v-Ki-ras2 Kirsten rat sarcoma 2 viral oncogene homolog	KRAS2	NM_004985	7.79
absent in melanoma 1-like	AIM1L	NM_017977	6.79
protein phosphatase 2, regulatory subunit B, beta isoform	PPP2R2B	NM_004576	6.75
integral membrane protein 2A	ITM2A	AL021786	6.43
cannabinoid receptor 1 (brain)	CNR1	U73304	6.09
cholinergic receptor, nicotinic, alpha polypeptide 5	CHRNA5	NM_000745	6.02
SMC5 structural maintenance of chromosomes 5-like 1 (yeast)	SMC5L1	NM_015110	5.78
protein kinase, cAMP-dependent, regulatory, type I, beta	PRKAR1B	AL833563	5.77
ATP-binding cassette, sub-family A (ABC1), member 1	ABCA1	AF285167	5.36
**Down-regulated at 24 h post-treatment**			**(From 0.13 to 0.37-fold)**
interferon-inducible protein p78 (mouse)	MX1	NM_002462	0.13
interferon-induced protein 44	IFI44	NM_006417	0.16
interferon, alpha-inducible protein (clone IFI-15K)	G1P2	NM_005101	0.20
protein kinase, interferon-inducible double stranded RNA dependent	PRKR	NM_002759	0.21
2'-5'-oligoadenylate synthetase 2, 69/71 kDa	OAS2	NM_016817	0.24
BCL2-like 11 (apoptosis facilitator)	BCL2L11	NM_006538	0.28
methionyl aminopeptidase 2	METAP2	NM_006838	0.28
signal transducer and activator of transcription 1 (91 kDa)	STAT1	NM_007315	0.29
myxovirus (influenza virus) resistance 2 (mouse)	MX2	NM_002463	0.30
SP110 nuclear body protein	SP110	AF280094	0.30
sialoadhesin	SN	NM_023068	0.30
mitochondrial ribosomal protein S14	MRPS14	NM_022100	0.31
similar to *S. cerevisiae *SSM4	TEB4	NM_005885	0.31
XIAP associated factor-1	HSXIAPAF1	NM_017523	0.33
SP110 nuclear body protein	SP110	NM_004509	0.35
retinitis pigmentosa 2 (X-linked recessive)	RP2	NM_006915	0.36
zinc finger protein 83 (HPF1)	ZNF83	NM_018300	0.36
zinc finger antiviral protein	ZAP	NM_020119	0.36
TIA1 cytotoxic granule-associated RNA binding protein	TIA1	NM_022173	0.38
RAN binding protein 9	RANBP9	NM_005493	0.38

Top 20 genes up- or down-regulated in human immature DCs in response to treatment with cichoric acid (50 g/ml) of *Echinacea purpurea *for 4 h (A) by fold change in expression.

**Table 3:** Genes showing overlapping up- or down-regulation in iDCs after treatment with [BF/S+L/Ep] or cichoric acid

**Genes up-regulated**	**Genebank**	**Fold Change in Treatment with [BF/S+L/Ep] (From 23.9 to 2.7-fold)**	**Fold Change in Treatment with Cichoric Acid (From 14 to 3.1-fold)**
adaptor-related protein complex 4	NM_007077	23.91	14.10
secreted frizzled-related protein 1	NM_003012	10.17	10.11
cholinergic receptor	NM_000745	9.95	6.02
chorea acanthocytosis	AB023203	9.91	9.56
tripartite motif-containing 45	NM_025188	8.34	8.26
zinc finger protein (ZFD25)	NM_016220	5.27	5.35
SUMO-1-specific protease	NM_015571	5.02	4.56
nuclear respiratory factor 1	NM_005011	4.97	3.39
pleckstrin homology-like domain	NM_007350	4.79	3.36
protein phosphatase 2	NM_004576	4.56	6.75
chromosome 19 open reading frame 2	NM_003796	3.91	3.27
SMC5 structural maintenance of chromosomes 5-like 1 (yeast)	NM_015110	3.87	5.78
myeloid/lymphoid or mixed-lineage leukemia	NM_004641	3.70	5.34
aryl hydrocarbon receptor interacting protein-like 1	NM_014336	3.60	3.70
left-right determination, factor B	NM_020997	3.53	4.14
ATP-binding cassette, sub-family A	NM_005502	3.35	5.36
RBP1-like protein	NM_016374	3.07	3.26
solute carrier family 26, member 10	NM_133489	2.89	3.05
insulin induced gene 1	NM_005542	2.87	3.12
ceroid-lipofuscinosis, neuronal 8	NM_018941	2.72	3.10
**Genes down-regulated**	**Gene bank**	**(From 0.08 to 0.38-fold)**	**(From 0.13 to 0.4-fold)**
Interferon-inducible protein p78 (mouse)	NM_002462	0.09	0.13
Interferon-inducible protein 44	NM_006417	0.16	0.16
Interferon, alpha-inducible protein (clone IFI-15K)	NM_005101	0.19	0.20
Mitochondrial ribosomal protein S14	NM_022100	0.27	0.31
2'-5'-oligoadanylate synthetase 2, 69/71 kDa	NM_016817	0.27	0.24
Myxovirus (influenza virus) resistance 2 (mouse)	NM_002463	0.29	0.30
XIAP associated factor-1	NM_017523	0.30	0.33
SP110 nuclear body protein	NM_080424	0.33	0.35
Signal transducer and activator of transcription 1, (91 kDa)	NM_007315	0.33	0.29
zinc finger antiviral protein	NM_020119	0.34	0.36
Solute carrier family 35	NM_012243	0.36	0.38

Human immature DCs were treated for 24 h with test samples and assayed for differential gene expression.

### Putative signaling networks/pathways involved in the modulatory effect of [BF/S+L/Ep] on iDCs

**Figure 4:**
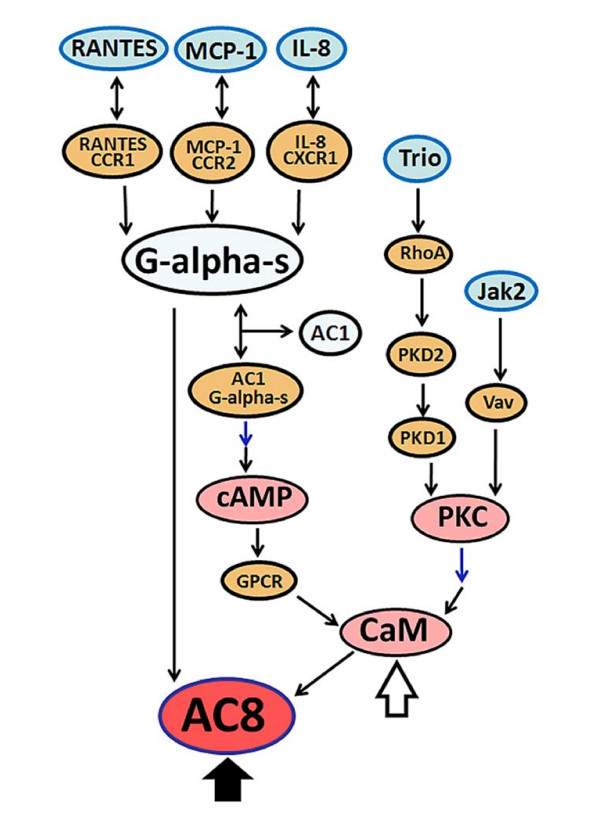
**Bioinformatics analysis of candidate molecular signaling networks of genes differentially expressed in [BF/S+L/Ep]-treated iDCs.** The TRANSPATH Professional 7.1 database was searched to assess the possible signaling pathways, networks or potential interactions among the responsive genes/target molecules in iDCs treated with [BF/S+L/Ep]. The 20 genes that were up- or down-regulated at least 5-fold over controls were analyzed. Specifically, connections (hits) within 7 genes were employed as the parameter for the current search. Two postulated key molecules/pathways, Adenylate cyclase (AC8) and calmudulin (CaM), responsive to [BF/S+L/Ep] treatment were indicated indicated by thick and thin arrows, respectively.

To identify the possible putative signal transduction pathways, we analyzed, for 4 hr and 24 hr treatments, the top 20 up-regulated genes in human iDCs in response to treatment with [BF/S+L/Ep] using TRANSPATH soft ware. Signal transduction pathways involving the MCP-1, IL-8, CCL5, JAK2 and TRIO genes with ≥ 5-fold change in expression on treatment (Table 1A) was identified. Signaling pathway networking and gene function analyses led to the hypothesis that treatment of iDC with [BF/S+L/Ep] may activate the cyclic AMP and PKC pathways leading to the regulation of a key down stream molecule, adenylate cyclase 8 (AC8), in a Ca^2+ ^dependent manner (Figure 4).

### Construction of proteome maps and identification of differentially expressed proteins in human iDCs

**Figure 5:**
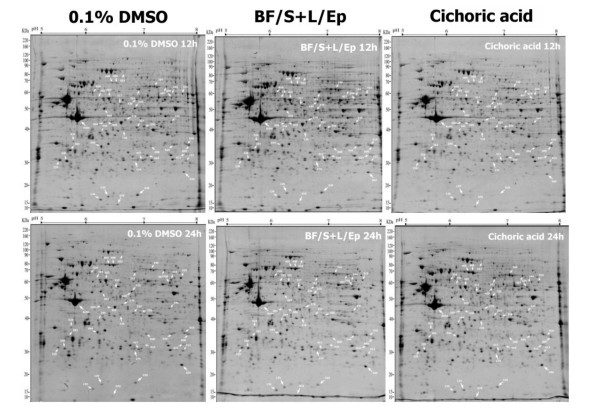
**Differential expression pattern of proteins in iDCs treated with [BF/S+L/Ep] and derived phytocompounds. **Human iDCs in culture were treated with 0.1% DMSO (vehicle control), [BF/S+L/Ep] (75 μg/ml) and cichoric acid (50 μg/ml) for 12 or 24 h. Aliquots of equal amounts of solubilized total proteins from various test cell samples were analyzed by 2-D gel electrophoresis and MALDI-TOF-MS. Approximately 1,300 protein spots were detected in iDCs. Seventy gels were used for the present study, and highly similar results were obtained from three independent experiments, unless indicated otherwise.

**Figure 6:**
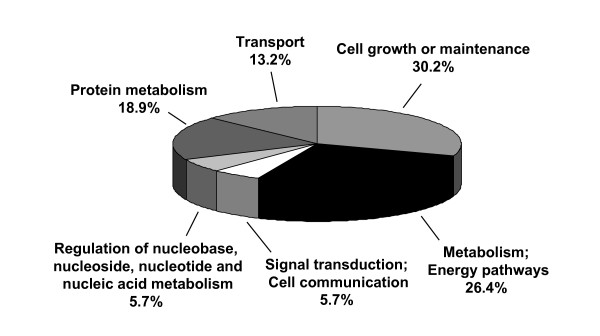
**Grouping of the identified human iDC proteins expressed in response to treatment with [BF/S+L/Ep], according to their known cellular or biochemical functions.** Assignment of biochemical functions of each protein involved with the use of information from the Swiss-Prot database at the ExPASY Molecular Biology Server .

Treatment of iDCs with 75 μg/ml [BF/S+L/Ep] or 50 μg/ml cichoric acid resulted in differential expression of 43 proteins (Figures 5 and Table 4) identified by MALDI-TOF-MS. These 43 proteins were further categorized by function with use of the Swiss-Prot database. Most of the proteins responding to [BF/S+L/Ep] (30.2%) were cell growth- or maintenance-related proteins (Figure 6); these included structural and cytoskeleton proteins such as macrophage-capping protein, cofilin, profilin, F-actin capping protein β subunit, and laminin A/C (Table 4). Other major classes were energy pathway-related proteins or metabolic enzymes (26.4%), proteins involved in protein metabolism or degradation (18.9%), and ion channel/transport proteins (13.2%). A small number were related to signal transduction and cell communication (5.7%), and others were associated with the regulation of nucleotides and nucleic acid metabolism (5.7%). Some proteins of interest include oxidative stress-related proteins (superoxide dismutases [Mn, Cu and Zn], catalase and peroxiredoxin 6) and cytoskeleton related proteins (gelsolin, macrophage capping protein, actin related protein 3, coronin-1C, beta adducin, vimentin, WD-repeat protein 1, T-complex protein 1 epsilon subunit, T-complex protein 1- beta subunit, cofilin, F-actin capping protein beta subunit 2, actin-related protein 2/3 complex subunit 2, profilin-1). These specific proteins may warrant further evaluation because of their apparent roles in modulation of DC maturation and function.

**Table 4:** Up- or down-regulation of proteins in human iDCs in response to treatment with [BF/S+L/Ep] or cichoric acid

**Protein Name**	**Protein Expression Ratio: Phytocompound Treated/Control (in 0.1% DMSO, Untreated)**				**Accession Number (SwissProt Data Base)**
	**[BF/S+L/Ep] (75 ug/ml)**		**Cichoric Acid (50 ug/ml)**		
	**12 h**	**24 h**	**12 h**	**24 h**	
**Up-regulation in protein expression**					
Gelsolin	1.12	1.1	2.37	5.09	P06396
Macrophage capping protein	1.39	1.13	1.31	1.34	P40121
Actin-related protein 3	0.97	1.04	1	1.23	P61158
Coronin-1C	2.55	0.71	1.74	1.1	Q9ULV4
Beta adducing	1.1	1.49	1.34	1.69	P35612
Rho GDP-dissociation inhibitor 1	0.94	1.16	0.83	1.49	P52565
Rab GDP dissociation inhibitor beta	0.9	1.37	0.88	1.46	P50395
T-complex protein 1, epsilon subunit	1.29	1.03	1.17	1.44	P48643
Serum albumin [Precursor]	1.24	1.1	1.23	0.87	P02768
Vimentin	2.44	0.77	1.1	0.7	P08670
Protein disulfide-isomerase A3 [Precursor]	1.09	1.21	0.93	1.03	P30101
Carbonic anhydrase II	1.76	1.16	1.32	1.01	P00918
Catalase	1.14	1.21	1.28	1	P04040
Superoxide dismutase [Mn], mitochondrial	1.72	1.23	1.09	0.84	P04179
Peroxiredoxin 6	1	1.13	1.1	1.31	P30041
Biliverdin reductase A [Precursor]	1.21	1.05	1.32	1.27	P53004
Poly(rC)-binding protein 1	0.87	1	0.79	1.31	Q15365
WD-repeat protein 1	1.54	1.17	1.74	1.47	O75083
TFIIH basal transcription factor complex helicase subunit	0.88	1.06	0.86	1.36	P18074
Chloride intracellular channel protein 1	0.98	1.04	0.94	1.32	O00299
Potassium voltage-gated channel subfamily H member 5	0.89	1.07	1.14	1.68	Q8NCM2
**Down-regulation in protein expression**					
Cofilin, non-muscle isoform	0.75	0.84	0.09	0.42	P23528
F-actin capping protein beta subunit	0.99	1.09	0.87	1.23	P47756
Actin-related protein 2/3 complex subunit 2	0.83	0.53	0.94	0.58	O15144
Profilin-1	0.84	0.76	0.6	0.51	P07737
Lamin A/C	0.81	0.69	0.45	0.76	P02545
Phosphoglycerate mutase 1	1.3	0.83	0.89	1.76	P18669
Superoxide dismutase [Cu-Zn]	0.84	0.99	0.78	1.48	P00441
Dihydrolipoyl dehydrogenase, mitochondrial [Precursor]	0.97	0.61	1.12	0.82	P09622
Galactose mutarotase	1.08	1.05	1.32	1.46	Q96C23
Delta3, 5-delta2, 4-dienoyl-CoA isomerase, mitochondrial [Precursor]	1.24	0.84	1.23	0.22	Q13011
Dihydropyrimidinase related protein-2	0.96	1.22	0.77	1.74	Q16555
26S proteasome non-ATPase regulatory subunit 13	0.94	1.32	0.95	1.42	Q9UNM6
60S acidic ribosomal protein P0	1.07	1.02	0.7	1.13	P05388
Heterogeneous nuclear ribonucleoprotein H	1	0.7	0.74	0.52	P31943

### Confirmation of up- or down-regulation of specific proteins

**Figure 7:**
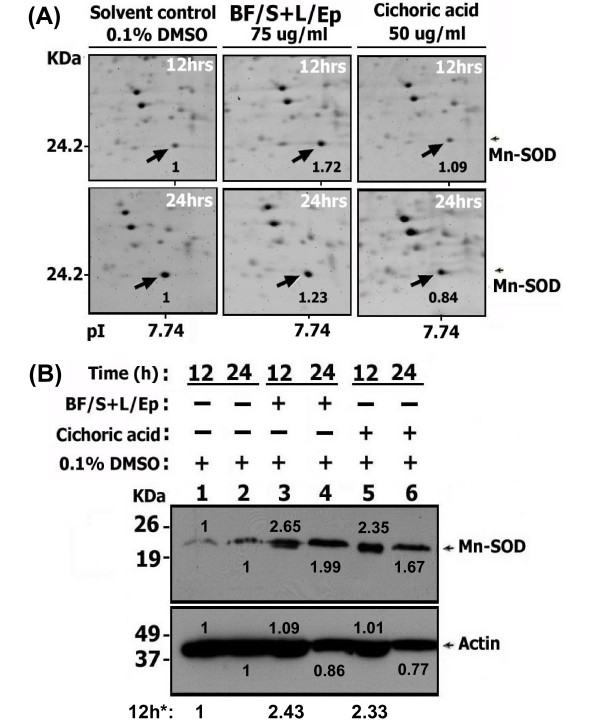
**Confirmation of the 2-D gel proteomic results on up-regulation of Mn-SOD protein expression with Western blot analysis.** (A) Differential expression of Mn-SOD protein in iDCs treated with [BF/S+L/Ep] (75 μg/ml), cichoric acid (50 μg/ml) or vehicle control, at both 12 and 24 h post-treatment, as revealed by 2-D gel electrophoresis, SYPRO Ruby dye staining, and quantification by PDQuest analysis. The arrows indicate the Mn-SOD protein spots detected in different treatments. The expression ratio compared to control gel is shown in black. (B) Western blot analysis of expression of Mn-SOD and actin in human iDCs treated with phytocompounds. Total cellular proteins were extracted and separated by 10% SDS-PAGE, and Western blot analysis performed with anti-Mn-SOD or anti-actin antibodies. Actin was used as a sample-loading control. Similar results were obtained from three independent experiments.

**Figure 8:**
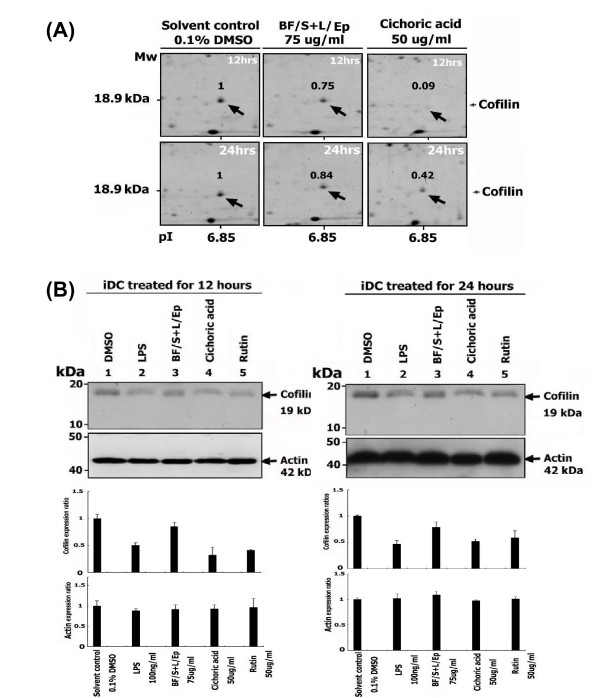
**Confirmation of the 2-D gel proteomic results on down-regulation of cofilin protein expression with Western blot analysis.** (A) Differential expression of cofilin in human iDCs treated with [BF/S+L/Ep] (75 μg/ml), cichoric acid (50 μg/ml) or vehicle control, at 12 and 24 h treatment, as revealed by 2-D gel electrophoresis. (B) Western blot analysis of expression of cofilin in human iDCs. Other experimental details are the same as described in Figure 7. Similar trends were observed in three independent experiments.

Western blot analysis was conducted to confirm some of the up-regulated proteins observed from proteomic analysis. The expression of Mn-SOD (SOD2) was increased 2.65- to 1.99-fold with [BF/S+L/Ep] and 2.35- and 1.67-fold with cichoric acid treatment at 12 and 24 h, respectively, as compared with vehicle controls, which is consistent with results from 2-D gel electrophoresis (Figure 7, Table 4). The levels of cofilin determined by 2-D gel electrophoresis (Figure 8A) were slightly decreased, by 0.75- and 0.84-fold, with [BF/S+L/Ep] and greatly decreased, by 0.09- and 0.42-fold, with cichoric acid treatment at 12 or 24 h, respectively. Interestingly, confirmation by Western blot analysis revealed only a 0.5- and 0.32-fold decrease in cofilin level at 12 and 24 h with cichoric acid treatment (Figure 8B). Both assay systems revealed reduced effect of [BF/S+L/Ep] on cofilin expression in iDCs (0.75- and 0.84-fold, respectively) (Figure 8A Vs 8B). Nevertheless, these results confirmed our finding that the expression of cofilin in iDCs can be substantially reduced by treatment with cichoric acid and may be slightly inhibited by [BF/S+L/Ep]. Rutin, another major index component of the [BF/S+L/Ep], effectively inhibited the expression of cofilin, by 0.4- and 0.6-fold at 12 and 24 h, respectively, in iDCs (Figure 8B). Treatment with 100 ng/ml lipopolysaccharide (LPS, used as a positive control) conferred an approximately 0.5- to 0.4-fold decrease in cofilin expression as compared to controls (Figure 8B).

## Discussion

Results of some clinical studies are controversial about the effects of the traditional herbal medicine *Echinacea *extracts [14-19], even though recent studies seemed to provide some support for its possible beneficial effects for treatment of the common cold [20,21]. In addition, studies from animal experiments have shown that alkamides form *Echinacea *extracts may confer immune-modulatory activities [35,36]. Among these different observations, we believe it is important to evaluate systematically the specific and multiple effects of *Echinacea *phytocompounds on human immune cells, at the cellular and molecular levels. Previously, we reported that un-fractionated, crude extracts of *E. purpurea *plant tissues could affect specific cell-surface markers of human DCs [25], which suggests the potential of using human DCs as an experimental system for in-depth study of the effect of *Echinacea *phytocompounds on important human immune cell systems. In the current study, we evaluated and characterized the possible immune-modulatory effect(s) of phytocompounds from *E. purpurea*, as an organic solvent-fractionated phytocompound mixture or a single major component (cichoric acid), on human DCs. Using transcriptome and proteome experimental approaches, we analyzed the global and differential gene expression patterns at the mRNA and protein levels in human monocyte-derived iDCs treated with test phytocompounds.

Previous studies have demonstrated that DCs play important roles in initiating and regulating innate as well as adaptive immunities [22]. Under normal conditions, DCs are present in most tissues in a relatively immature state, but with inflammation, irritation or danger signals from foreign antigens or environment, iDCs undergo rapid changes and initiate a cascade of activities to defend the body system. The phenotypic and functional characteristics of DCs are intimately and dynamically linked to their stage of differentiation and maturation [37]. Human DCs have been recently recognized as a promising cell system for *ex vivo/in vitro *tissue culture manipulations for potential clinical application as tumor vaccines for cancer therapy [23].

Initially, we used the expression of a highly specific cell-surface and DC maturation maker, CD83, as a key indicator for assessing possible immune modulatory effects of *Echinacea *extracts on human DCs. The butanol fraction [BF/S+L/Ep] significantly stimulated but the ethylacetate (EA) fraction greatly inhibited CD83 expression in iDCs, which suggests a possible immune modulatory effect of [BF/S+L/Ep] on the maturation of iDCs and that different sub-fractions of the phytocompound mixtures/herbal extracts could confer different or even opposite biological activities. These findings thus strongly suggest the urgent need for a clear, specific and stringent definition(s) of the extraction, fractionation and plant-tissue usage of the medicinal herb preparations and the derived phytocompounds for both laboratory experimental studies and commercial applications. The lack of such definition and information has already raised public concern.

Since our results on CD83 protein expression suggested that the [BF/S+L/Ep] may stimulate the maturation of iDCs, we subjected this butanol fraction to HPLC analysis to characterize the major components and some possible active compounds. We identified seven specific phytocompounds, namely hypoxanthine, caffeic acid, chlorogenic acid, cichoric acid, rutin, quercetin 3-*O*-rhamnosyl-(1→6)-galactoside, and kaempferol 3-*O*-rhamnosyl-(1→6)-galactoside from the [BF/S+L/Ep] fraction. The content of two major compounds cichoric acid **(4) **and rutin **(7) **were quantified, and this data could be used as chemical fingerprints and index compounds for future study and standardization of the bioactive [BF/S+L/Ep] fraction of *E. purpurea*. Potential future application of the [BF/S+L/Ep] fraction for use as remedy, nutraceutical or food supplement would have to wait until future studies are carried out with the considerations of appropriate dosage, bio-activity and defined immuno-modulatory functions to be assayed under *in vivo *conditions.

Use of the Affymetrix microarray analysis revealed approximately 10% of the whole human genome effectively expressed in DCs (data not shown). A total of 400 genes (≈18% of the expressed genes) were differentially or specifically regulated during the differentiation or maturation of iDCs. The top 20 genes with significantly up regulated expression (> 3-fold change) at 4 h post-treatment with [BF/S+L/Ep] include some important early-responsive chemokine genes such as CCL2, CCL5 and IL-8, previously reported to play important roles in inflammatory responses [38]. Although treatment with cichoric acid did not affect CD83 expression, as was observed for [BF/S+L/Ep], it nonetheless up-regulated a similar subset of early response genes. Additional analysis of genes with overlapping expression on treatment with both [BF/S+L/Ep] and cichoric acid revealed that some of the interferon-inducible proteins, including IFI44, IFI78 (Table 3), IFIT1 and the interferon alpha-inducible protein (Table 1B), were all down-regulated at 24 h post-treatment. Thus, several key chemokine genes are likely involved in the early response to treatment with specific *Echinacea *phytocompounds, and another set of interferon-inducible genes belong to the group of late-responsive genes. We also performed gene-clustering analysis to classify the gene expression patterns in human iDCs. A total of 382 genes were grouped into six clustering sets on the basis of their expression in response to [BF/S+L/Ep] (Figure 3). In contrast, cichoric acid and vehicle treatment produced no clustering differences, which suggests the lack of a multi-domain or substantial effect of cichoric acid on most expressed genes in iDCs.

We have identified a hypothetical signaling network involving [BF/S+L/Ep]-induced early response genes such as CCL2 (MCP-1), IL-8, CCL5, JAK2 and TRIO (Figure 4). This signaling network revealed possible activation of cAMP and PKC pathways leading to the regulation of AC8 through a Ca^2+ ^receptor, calmodulin. Previous studies have demonstrated that activation of cAMP induces DC maturation and migration [39]. PKC plays an important role in DC differentiation and antigen presenting function [40]. In addition, Ca^2+ ^dependent pathways have been shown to regulate DC maturation and migration [41]. Our results on increased expression of CD83 maturation marker coupled with candidate signal transduction pathways strongly suggest that [BF/S+L/Ep] may enhance the iDC maturation and function, which warrants further systematic investigation.

2-D gel electrophoresis revealed 100 of the 1,300 detectable protein spots (≈7.7%) affected in iDCs after 12- or 24-h treatment with [BF/S+L/Ep]. We suggest that this 7.7% change in iDC protein levels may reflect a marked change in protein expression, since the other cell types we have studied in parallel (e.g., primary T-cells) showed only a 3% to 5% change (data not shown). We observed significant up-regulation of antioxidant defense enzymes such as Mn-SOD, catalase and peroxiredoxin 6 in iDCs in response to [BF/S+L/Ep] and cichoric acid. Endogenous antioxidants have been reported to play key role in dendritic cell survival, and ability to induce T cell activation and regulate the polarity of immune responses [42,43]. Several cytoskeletal and actin-binding proteins (Table 4) were significantly up-regulated in [BF/S+L/Ep] or cichoric acid treated iDCs. In DC, the changes in actin cytoskeleton components are essential for the formation of its characteristic dendrites and veils, as well as an immunological synapse necessary for antigen presentation [44]. In addition, actin cytoskeleton rearrangement is important for the motility and migration of cells and may influence the migration of DC to lymph nodes during their maturation [45]. Results from 2-D gel electrophoresis (Figures 7A and 8A) and Western blot analyses (Figures 7B and 8B) confirmed in general the trend of changes in differential expression of Mn-SOD and cofilin. We therefore hypothesize that a specific group of phytocompounds in *Echinacea *may turn on the antioxidant defense system and regulate the rearrangement of cytoskeleton, which in turn may contribute to the positive enhancement of the immunemodulatory activities of DCs.

The present study has identified a pool of known or unknown genes associated with the differential expression of a spectrum of cytokines and chemokines and other immune cell activities in response to treatment with *Echinacea *compounds in DCs (Tables 1 to 4, and Figure 3). In addition, changes in level of molecules related to cell adhesion, immune-response and antigen presentation were observed in this human DC model, providing candidate target genes/proteins for future cross-talk studies of the biology of human DCs. These differentially expressed genes and proposed candidate signaling pathways/networks obtained via bioinformatics approaches have provided us with useful potential clues and molecular targets for future studies of molecular mechanisms underlying specific immune modulatory effect(s) of important medicinal herb *Echinacea purpurea *and derived phytocompounds in iDCs.

## Conclusion

Since dendritic cells are well characterized and recognized as potent antigen presenting cells and have been shown to play critical roles in both innate and adaptive immunities, we have employed human DCs for functional genomics studies to evaluate possible immuno-modulatory effects of specific phytocompound mixtures from a popular medicinal herb extract, *Echinacea purpurea*. Specific and differential gene expression patterns were detected in time-course experiments of iDCs treated with the bioactive phytocompound fraction ([BF/S+L/Ep]) and its major index compound (cichoric acid). Transcritome analyses showed significant up regulation of specific chemokine and cytokine genes, including CXCL2, CCL5, CCL2, IL-8, IL-1β and IL-18, within 4 h after [BF/S+L/Ep] treatment of iDCs. Bioinformatics studies suggested that a key molecular signaling network involving a number of the detected immune-modifier genes may lead to the activation of a downstream adenylate cyclase 8 activities, contributing to the observed cellular and molecular activities in test cells. Subsequent proteomic studies further showed that expression of specific antioxidation and cytoskeletal proteins, known to play important cellular physiological roles in DCs, are increased in cichoric acid or [BF/S+L/Ep]-treated cells. Based on these results, we conclude from this study that specific phytocompound mixtures present as major or active components in the test traditional herb may confer defined and significant immuno-modulatory effects on specific immune cell types. The candidate target molecules and molecular signaling mechanisms identified from this and previous study [25] provide us with useful information and hypothesis for future studies on characterization of human DCs in response to treatment with medicinal phytocompounds at the transcriptome and proteome levels.

## Methods

### Plant materials and crude extract preparations

*E. purpurea *plants at flowering stage were harvested from a reputable organic farm in Puli, Nantou County, Taiwan. Stem and leaf (S+L) tissues of fresh plants were extracted at room temperature by imbibition with 70% aqueous ethanol as previously reported [25]. The total 70% ethanolic extracts were concentrated (1 L) and successively partitioned with ethyl acetate (1 L × 3 times) and *n*-butanol (1 L × 3 times) to yield three sub-fractions, designated as the EA, BuOH and Water fractions. The percentage of yield for the three fractions was calculated as 10.3%, 7.23%, and 77.2%, respectively, of the 70% ethanolic extracts in dry weight.

### Generation of human dendritic cells

Monocyte-derived dendritic cells were generated from peripheral blood mononuclear cells (PBMCs) isolated from healthy volunteers blood samples as described previously [25]. Briefly, CD14^+ ^monocytes were isolated by passing the PBMCs through a magnetic cell separation system (Miltenyi Biotec, Germany). CD14^+ ^cells at 95% purity were cultured for 7 days in AIM-V medium in the presence of GM-CSF (1000 U/ml) and IL-4 (500 U/ml) to obtain immature dendritic cells (iDCs).

### Flow cytometry

At 24 h after treatment with *E. purpurea *crude extract, the S+L extract, or the three subfractions, iDCs were harvested and analyzed by immunofluorescence staining. Monoclonal antibodies labeled with fluorescent dyes i.e., HLADR-FITC, CD14-PE, CD32-PE, and CD83-FITC from Immunotech (Fullerton, CA) and CD86-FITC from Pharmingen (Fullerton, CA) were used. After incubation with specific antibodies at 4°C for 30 min, cells were washed twice with PBS and fixed with 1% paraformaldehyde, and subjected to analysis using a Coulter EPICS XL flow cytometer (Beckman/Coulter, Durham, NC).

### Cell viability assay

Human iDCs (4 × 10^5 ^cells/ml) were incubated with 0.1% DMSO (vehicle control), test plant extract or derived phytochemicals in basal medium in 96-well plates for 24 h in a 5% CO_2 _incubator. All treatments were performed in triplicate. Viability of iDCs was determined by MTT assay, and percentage cell viability was calculated as previously described [25]. The derived plant extracts from *E. purpurea *were examined by a Limulus Amebocyte Lysate (LAL) test (E-TOXATEâ Kits, Sigma, MO, USA) for detection of possible endotoxin (LPS) contamination before bioactivity assays.

### Phytocompound isolation

The BuOH fraction of the S+L tissues of *E. purpurea*, designated as [BF/S+L/Ep] hereafter, was subjected to chomatography on RP-18 silica gel with the H_2_O/MeOH solvent system to yield four fractions. Fraction 1 was further purified by filtration though a Sephadex LH-20 (Amersham Pharmacia Biotech) column with a H_2_O/MeOH gradient solvent system to yield three sub-fractions. RP-18 high-performance liquid chromatography (HPLC) column (Phenomenex Luna, 5 μm C18, 250 × 10 mm) with a solvent system, 0.05% TFA/H_2_O (solvent A) and 0.05% TFA/MeOH (solvent B) and gradient profile (5-5% for 0 to 5 min, 5-20% for 5-30 min, and 20-50% for 30-60 min) was used for isolation of single compounds. Quantification of the index compounds in the active [BF/S+L/Ep] fraction involved HPLC. The peak areas corresponding to the content of candidate compounds of a known concentration of the active fraction were calculated on the basis of a standard calibration curve of the individual index compounds. The HPLC profile and the relative content of the index compounds were routinely used for quality control.

### Chemical fingerprinting of the active [BF/S+L/Ep] fraction using LC/MS

LC/MS was employed to analyze the chemical fingerprint of the [BF/S+L/Ep] fraction. LC/MS was carried out using a ThermoFinnigan LCQ Advantage ion trap mass spectrometer equipped with an Agilent 1100 series liquid chromatographer, at the Metabolomics Core Facility of the Agricultural Biotechnology Research Center, Academia Sinica, Taiwan. Aliquot of a 5 μl sample of [BF/S+L/Ep] (10 mg/ml) was injected and analyzed at a flow rate of 0.2 ml/min. HPLC profiling involved an RP-18 column (Phenomenex Luna, 3 μm C18, 150 × 2 mm) and eluted compounds detected by UV absorbance at 254 and 330 nm before MS analysis. The solvent gradient for HPLC was 0.05% TFA/H_2_O (solvent A) and 0.05% TFA/MeOH (solvent B): 5–5% for 0 to 5 min, 5–20% for 5–30 min, 20–45% for 30–40 min, and 45–50% for 40–50 min). Ionization involved the positive ion mode for all analyses.

### DNA microarray analysis of differential gene expression patterns

Total RNA was isolated by use of TRIzol^® ^Reagent (Invitrogen) according to the manufacturer's instructions to generate cRNA targets. A total of 7 μg of RNA from each sample was used to synthesize the first-strand cDNA with T_7_-Oligo (dT) primer and T7 RNA polymerase by *in vitro *transcription reaction. The biotinylated cRNA products were then cleaned according to the Affymetrix protocol. Aliquots of 15 μg of total cRNA per sample were then hybridized to the Affymetrix gene chip HG-U133A containing approximately 22,283 probe sets following the manufacturer's instructions. Images were obtained by use of standard Affymetrix scanners. The cell intensities and detection from the nine hybridized oligonucleotide microarrays were derived by use of the Affymetrix Microarray Suite 5 (MAS 5.0) with default settings. Normalization was by dChip with the PM-MM model-based approach to obtain the expression indices for each probe set [46]. The pool of the replicate arrays, CM-00h-1 and CM-00h-2, was used as a baseline for normalization and was applied to the calculation of log2 ratios, M, for the test arrays. Comparative expression analysis of the two arrays by the MAS 5.0 system reported a level of "no change in gene expression pattern" of 98.0% (with a correlation of 0.9916). This high reproducibility of results indicates very high reliability and very low variation of our experimental data obtained from the in-house Affymetrix system. The gap statistic and K means analysis were used to distinguish gene clusters as described previously [47,48]. The putative signal transduction pathways were analyzed using TRANSPATH Professional 7.1 (BIOBASE Biological Databases, Germany). The microarray data obtained from this study have been deposited to the Gene Expression Omnibus database at NCBI (GEO; ) under the accession number GSE12259.

### 2-D gel electrophoresis and image analysis

Total cellular proteins of iDCs were extracted and prepared in 0.3 ml of lysis buffer [7 M urea, 2 M thiourea, 4% 3,3-cholamidopropyl-dimethylammonio-1-propanesulfonate (CHAPS), 10% 1,4-dithiothreitol (DTT), 2% Pharmalyte pH3-10] by vortexing for 1 h, and protein supernatants were collected after ultracentrifugation at 55,000 rpm for 1 h. Protein concentrations were determined using Bio-Rad BCA protein assay kit (Bio-Rad, Hercules, CA). 2-D gel electrophoresis involved the PROTEAN isoelectric focusing and electrophoresis system (Bio-Rad, Hercules, CA) with modification [49]. Briefly, 550 μg of protein was diluted in 300 μl of rehydration buffer containing 7 M urea, 2 M thiourea, 4% CHAPS, 2% 1,4-dithioerythitol, 0.13% Biolyte 5–8 (Bio-Rad) and 0.07% Biolyte 8–10 (Bio-Rad). Protein samples were loaded onto IPG strips (Bio-Rad; 17 cm, pH 5–8) through passive rehydration overnight. Proteins underwent electro focusing analysis in slow-ramping mode with a final voltage of 8,000 V for a total of 25,000 Vh. Following a two-step equilibration, IPG strips were processed by 10–16% gradient SDS-PAGE and electrophoretic separation at 200 V. Gels were then fixed and stained with SYPRO-Ruby fluorescent stain (Molecular Probes; Eugene, OR) according to the manufacturer's instructions. Stained gels were visualized by a Typhoon scanner system (Amersham). Digitalized gel images were analyzed by 2-D PDQuest, V.T.O. (Bio-Rad). Protein spots on gels were automatically detected with use of the PDQuest system. The 2-D gel images of iDCs proteins obtained with IPG strips (pH 5–8) were matched separately, with each match set containing 9 images, with 3 images for control replicates. The image with the highest number of spots was selected as the master gel. Automatically detected images of protein spots in test gels were then manually edited to include the low intensity spots and corrected for spot artifacts. For the match set containing images from pI 5–8, the spot volume (intensity integrated over the spot area) was normalized to the total volume of spots in the gel. Data were then exported to Microsoft Excel.

### Protein identification by MALDI-TOF-MS

Protein extraction was performed as previously described [50]. Briefly, each gel spot was cut into small pieces with a scalpel, washed with 700 μl of double distilled H_2_O, and subjected to reduction reaction by DTT. Alkylation reaction then carried out by adding 55 mM iodoacetamide for 1 h at room temperature in the dark. Gel spots were washed with ammonium carbonate then acetonitrile. Gel pieces were dried and then immersed in trypsin solution. The in-gel digestion was performed at 37°C overnight. Resulting tryptic peptides were extracted twice from the test gel into 70% acetonitrile/5% HCOOH by sonication then centrifugation. The combined supernatant was dried under a Savant Speed Vac, and 6 μl of 1% HCOOH was added to each test sample. Protein identification by MALDI-Q-TOF-MS [51] was performed at the Proteomics Core Facility of the Institute of Biological Chemistry, Academia Sinica. The MS data with monoisotopic peptide masses were searched against the NCBI protein database with use of the MASCOT search engine (Matrix Science; London, UK). Variable modifications, methionine oxidation and cysteine carbamidomethylation were taken into account in our data analyses.

### Western blot analysis

Total protein was isolated from test iDCs with ice-cold lysis buffer (150 mM NaCl, 0.5% Triton X-100, 50 mM Tris-HCl (pH 7.4), 20 mM EGTA, 1 mM dithiothreitol (DTT), and protease inhibitor cocktail tablets) for 10 min and centrifuged at 12,000 × *g *for 20 min. Protein samples were subjected to western blotting as described previously [52]. Briefly, the proteins were detected after overnight incubation at 4°C with 1:1000 dilution of Mn-SOD or cofilin polyclonal antibodies (Millipore, MA, USA). Equal protein loading was assessed using mouse β-actin (Sigma Chemical Co., St. Louis, U.S.A). The proteins were visualized with an enhanced chemiluminescence (ECL) detection kit (Amersham Pharmacia Biotech, Buckinghamshire, UK).

## Authors' contributions

CYW served as key experimenter and author of the draft manuscript. VS improved the clarity by critical revision and reorganization of the manuscript, and incorporated some parts of the discussion section. MTC designed, optimized and completed the 2D gel/proteome experiments. CCH performed metabolite profiling and compound identification. HMW, KCY, CHC & PIH performed bioinformatics analyses on DNA microarray data as a team. TNW introduced and built the proteome study system of the lab. LFS is Co-PI and Co-correspondent author of this project/manuscript. NSY is PI and principal author of the manuscript. All authors read and approved the final manuscript.
